# Early Marriage and Its Determinants among Married Reproductive Age Group Women in Amhara Regional State, Ethiopia: A Multilevel Analysis

**DOI:** 10.1155/2021/1969721

**Published:** 2021-03-08

**Authors:** Setognal Birara Aychiluhm, Ayenew Kassie Tesema, Abay Woday Tadesse

**Affiliations:** ^1^Department of Public Health, College of Medicine and Health Sciences, Samara University, Samara, Ethiopia; ^2^Department of Health Education and Behavioral Health, Institute of Public Health, College of Medicine and Health Sciences, University of Gondar, Gondar, Ethiopia

## Abstract

**Introduction:**

Amhara region has one of the highest rates of female child early marriage in Ethiopia, with eighty percent of girls in the region being married at the age of eighteen. Therefore, this study was intended to assess the prevalence and determinants of early marriage among women, in Amhara regional state.

**Methods:**

The data were extracted from the 2016 Ethiopian Demographic and Health Survey. The study included a sample of 2887 (weighted) married women from 645 clusters in Amhara regional state. The data were collected using a two-stage cluster design that includes the selection of enumeration areas as a first stage and selection of households as a second stage. A multilevel logistic regression model was fitted to determine the individual and community-level factors associated with early marriage.

**Result:**

The study revealed that 73% [95% CI 71.38, 74.62] of women aged 15–49 years were married before 18 years old. In the multilevel multivariable model; living as a rural dweller (AOR = 4.33; 95% CI: 2.17, 8.64), no education (AOR = 2.52; 95% CI: 2.23, 9.51), attending only primary education (AOR = 2.31; 95% CI: 1.68, 8.53), parental decision-maker when to get marriage (AOR = 3.44; 95% CI: 2.20, 5.39), being poorer (AOR = 1.38; 95% CI: 1.16, 4.83), and poorest wealth status (AOR = 2.37; 95% CI: 2.19, 7.83) were the independent predictors of early marriage.

**Conclusion:**

The prevalence of early marriage was high in Amhara region compared to other regions of the country. Therefore, the regional government should give due attention to access to education and encourage women's decision-making power upon the time of marriage especially those residing in rural parts of the region.

## 1. Introduction

Early marriage refers to “any marriage happens under the age of 18 years, where the girl is not ready for the marriage and childbirth” [1–3]. Over 700 million women were married before being 18 years old in the world [2]. Similarly, in Ethiopia, four in ten young women were married or get into union before the age of 18 and six million girls get married before the age of 15 [4]. Moreover, according to the Ethiopian Demographic and Health Survey 2000, 2005, and 2011 reports, more than two-thirds of married women reported that they were married before the age of 18 years [5–7]. Though the prevalence rate of early marriage varies from region to region in the country, eighty percent of girls in Amhara region got married before the age of eighteen which is higher than the national prevalence [8].

Early marriage has various consequences on the health and social outcomes of women and their children. These include increased risk of depression and suicidality; compromised sexual, reproductive, and maternal health [9–14]; greater risk of intimate partner violence [11, 13, 15]; decreased physical and social mobility; and decreased autonomy in decision-making within and outside the household [10, 16, 17]. Besides, early marriage also compromises girls' ability to attend a school that leads to school withdrawals [10, 16, 18–21]. Thus, it is a public health concern that violates international human rights laws and it seriously affects the development and health status of women and children [12, 17, 18, 20–27].

Studies conducted across the globe had identified various contributing factors of early marriage. These include family income, family size, educational level of the father and the respondent, young women who faced first sexual intercourse before 16, residence, wealth status, perceived ideal marital age, and media exposure [22, 28–33].

Different policies, strategies, and programs have been tried in the past decades to address the early marriage problems at global, and regional levels [1, 3]. Similarly, the revised Family Law of Ethiopia (FLoE) sets the legal age for marriage to be at 18 years and above [34]. However, the prevalence of early marriage in Amhara region is still consistently high [8, 35, 36]. There are some studies concerning early marriage undertaken in Amhara region in different settings [22, 29, 35]. However, most of these studies were limited in scope (that is the sample size and geographic area) and analyzed using single-level analysis, which does not consider the community-level factors. Moreover, using a single-level logistic regression analysis technique to analyze data that has a hierarchical structure nature (that is women nested within communities) violates the independence assumptions of regression [37, 38]. Hence, to address these limitations and to further estimate the significant effect of individual and community-level factors in the field of public health, this study used multilevel logistic regression analysis.

Therefore, the purpose of this study was to determine the prevalence and determinants (both individual and community-level factors) that are associated with early marriage in Amhara regional state, Ethiopia.

## 2. Methods and Materials

### 2.1. Study Area and Data Source

Amhara Regional State, one of Ethiopia's largest but most disadvantaged regions, is situated in the northwest and northcentral part of the country. The Amhara people (numbering approximately 20 million) comprise one of the nine ethnic divisions (regions) of Ethiopia and are predominantly (more than 85%) engaged in agriculture. The region is characterized by high levels of food insecurity due to recurrent drought and deforestation and low educational attainment (that is more than 60% of women over the age of 15 have never been to school) [5]. In this region, child marriage is rooted in religious and cultural traditions based around preventing a girl's honor, since sex before marriage is seen as an extremely shameful act. Therefore, a girl's worth is based on her virginity and her role of being a wife and a mother [39]. There was a large age difference between couples: nearly 75% of ever-married females were married to older men, and among these, the age difference was ten years or more in half of the cases. This age differential affects the level of communication, mutual understanding, and the balance of influence within the family [40]. Maintaining a family's dignity, social bonding, and desire to earn additional income “macha” (i.e., men's family pay money for the girl's family) are thought to be some of the main reasons to practice early marriage in the region [41].

The data for this study were retrieved from the DHS program official database website (http://dhsprogram.com), which was conducted in 9 regions and 2 city administrations of Ethiopia from January 18, 2016, to June 27, 2016 [10]. To conduct the 2016 EDHS, a two-stage stratified cluster sampling technique has been employed. In the first stage, enumeration areas were selected. In the second stage, 28 households per enumeration area were selected with an equal probability of systematic selection per enumeration area. A total of 645 EAs (202 in urban areas and 443 in rural areas) were selected with probability proportional to EA size. All women of reproductive age group were included in the first stage. All women aging 15-49 years who were either permanent residents of the selected households or visitors who stayed in the household the night before the survey were eligible to be interviewed [36]. For this study, 2887 (weighted) women of the reproductive age group in Amhara regional state were used for our analysis.

### 2.2. Study Variables

#### 2.2.1. Dependent Variable

Early child marriage is defined as a formal marriage or informal union before 18 years old [42]. It was categorized in such a way that 0 is marriage at age of 18 and above and 1 is marriage experienced before the 18th birthday.

#### 2.2.2. Independent Variables

The following are the independent variables:

1. Individual-related factors: who decide age at first marriage, women's education level, husband's education level, husband's occupation, wealth index, respondent's work status, religion, ethnicity, and exposure to any mass media
2. Community-related factors: residence site and cluster

### 2.3. Data Analysis

#### 2.3.1. Deceptive Statistics

The analysis was performed in R version 3.5.2 statistical software and STATA 15 statistical package. Based on the recommendation of EDHS, proportions and frequencies were estimated after applying sample weights to the data to adjust for disproportionate sampling and nonresponses, since the allocation of the sample in the EDHS to different regions and urban and rural areas were nonproportional. A detailed explanation of the weighting procedure can be found in the 2016 EDHS report [36]. Categorization was done for continuous variables using information obtained from different literatures, and recategorization was done for categorical variables accordingly to make it suitable for analysis.

#### 2.3.2. Multivariable Multilevel Analysis

To account the clustering effects (that is, women are nested within clusters) of 2016 EDHS data, a multivariable multilevel logistic regression analysis was applied to determine the effects of each predictor of early marriage. The basic data structure of the two-level logistic regression is a collection of *N* groups (cluster) and within-group *j* (*j* = 1, 2, 3, ⋯*N*), a random sample of *nj* of level-one units (individual women).

The response variable is denoted by the following: *Yij* = 1, if marriage happened under 18 years of age, and *Yij* = 0, if marriage happened 18 and above years of age.

In data with a nested structure like that of EDHS, the individual observations have some degree of correlation within a cluster because of common characteristics they share. Thus, when the correlation with the higher level is ignored and only the individual-level variables are considered, it might lead to a violation of the independence assumption between observations. This results in biased parameter estimates and will generally lead to underestimation of the standard errors and produce spurious significant results and accordingly to incorrect conclusions on effect sizes. In contrast, modeling group-to-group variation simultaneously with individual-to-individual variation in analysis has several advantages. It allows us to focus on the importance of both communities' and individuals' effects on individuals' health outcomes. By using the clustering information, it enables us to obtain statistically efficient estimates of regression coefficients [43]. Thus, to get the mixed effect (fixed effect for both the individual and community-level predictors and a random effect for the between cluster-variation), a multilevel analysis was applied for this analysis. Variables that were statistically significant at the bivariable multilevel logistic regression analysis were considered candidates for multivariable multilevel logistic regression analysis.

Finally, multivariable multilevel logistic regression analysis was performed to estimate the Adjusted Odds Ratios and to estimate the extent of random variations between communities.

#### 2.3.3. Model Building and Comparison

Four models containing variables of interest were fitted using R statistical database version 3.5.2.

Model I (empty model) was fitted without explanatory variables to test random variability in the intercept and to estimate the intraclass correlation coefficient (ICC) and Proportion Change in Variance (PCV). Model II examined the effects of individual-level characteristics, Model III examined the effect of community-level variables, and Model IV (full model) examined the effects of both individual and community-level characteristics simultaneously.

#### 2.3.4. Parameter Estimation Technique

In the multivariable multilevel models, the measures of association (fixed-effects) estimate the associations between the probability of early marriage and independent variables (individual and community-related) expressed as Adjusted Odds Ratio (AOR) with their 95% Confidence Intervals (CIs). The measures of variation (random-effects) were reported as intracluster correlation coefficient (ICC) which is the percentage variance explained by the higher level (community-level variables), and proportional change in variance (PCV) shows the change in the community-level variance between the empty model and the successive models [37, 44].

#### 2.3.5. Model Fitness

Akaike Information Criterion (AIC) was used to choose a model that best explains the data and the model with a low AIC value was taken. The AIC value for each subsequent model was compared and the model with the lowest AIC value was considered to be the best fit model [38, 45, 46]. Among the models considered, the full model has the smallest AIC value; therefore, the full model best fits the data. AOR with 95% Confidence Interval in the multivariable model was used to select variables that have a statistically significant association with early marriage.

## 3. Results

### 3.1. Socioeconomic and Demographic Characteristics of Respondents

Out of the total respondents, 2400 (83.12%) women were living in a rural site, 1938 (67.10%) of respondents were not attended formal education, and 356 (80.8%) of the respondents did not have media exposure towards early marriage (see Table 1).

### 3.2. Prevalence of Early Marriage

According to this study, overall 2098 (73% [95% CI: 71.38%, 74.62%]) were married before the age of 18 years in Amhara regional state. Of women who live in rural sites, 77.42% got married earlier 18 years of age and the rest were married 18 and above. Out of 2405 married women whose marriage was decided by their parents, 1,886 (78.44%) got married before the age of 18 years. The prevalence of early marriage in those who had no formal education was 1,529 (78.87%) (See Table 2).

### 3.3. Multilevel Logistic Regression

#### 3.3.1. Result of Empty Multilevel Logistic Regression Model

From the null model, variance of the random factor was 0.49 with 95% Confidence Interval of (0.32, 0.74), showing heterogeneous areas. Since the variance estimate is greater than zero, it indicates that there are enumeration (cluster) area differences in early marriage status among married reproductive age women in Amhara regional state, and thus, multilevel analysis should be considered an appropriate approach for further analysis.

The intraenumeration area correlation coefficient (ICC) indicated that 13% of the total variability in early marriage status is due to differences across cluster areas, with the remaining unexplained 87% attributable to individual differences. The Proportion Change in Variance (PCV) indicated that 10% of the variation in early marriage status across communities was explained by both individual and community-level factors included in the full model (see Table 3).

#### 3.3.2. Result of Multilevel Multivariable Logistic Regression Model (Full Model)

In the two-level mixed effect multivariable logistic regression model where both the individual and community-level factors were fitted simultaneously and when all the other independent variables are controlled, the odds of being married below the age of 18 in rural area is 4.33 more likely than living in an urban area (AOR = 4.33; CI: 2.17, 8.64).

After adjusting other covariates, the odds of being married below the age of 18 in the poorer and poorest level were 1.38 and 2.37 more likely to be early married compared to those women in the richest level (AOR = 1.38 and CI: 1.16, 4.83; AOR = 2.37 and CI: 2.19, 7.83), respectively.

Keeping other covariates constant, those women with no education were 2.51 more likely to be married below the age of 18 years than those who have higher education level (AOR = 2.51; CI: 0.71, 6.75) and those women with primary education level were 2.31 more likely to be married below the age 18 years compared to those with higher education level (AOR = 2.31; CI: 1.68, 8.53).

Regarding responsibility on who decides on age at first marriage, those women who have to decide by parents were 3.44 times more likely to have marriage below the 18 years than those who decide by themselves (AOR = 3.44; CI: 2.20, 5.39) (see Table 4).

## 4. Discussion

Previous studies were limited in scope and analyzed without considering the community-level related factors. This study is intended to determine the prevalence and determinants of early marriage among married reproductive age women in Amhara regional state using multilevel analysis.

This study revealed the prevalence of early marriage in Amhara regional state was 73% [95% CI: 71.38%,74.62%]. This prevalence is higher than a study conducted in Injibara, Ethiopia (44.8%) [29]. Moreover, the finding is also higher than those in studies conducted in Sudan (45.9%), India (22.6%), Sub-Saharan Africa (55%), and Roma of Serbia (50.4%) [28–31, 47].

In developing nations, there are geospatial variations in cultural practices and beliefs that strongly influence the acceptance and practices of early marriage [27, 48, 49]. Similarly, in Ethiopia, the existence of variations in sociocultural norms, values, and traditions encourages early marriage in Amhara region compared to other parts of the country [33]. Hence, this high prevalence could be attributed to conformity to social norms, seeking social status, ensuring virginity, material benefit (macha), and security for the future [35, 50, 51].

However, this prevalence is lower than the study conducted in east Gojjam, Ethiopia (87%) [35], and a study conducted in Bangladesh (78.2%). This discrepancy might be due to the small sample size in the previous studies compared to the current study.

In this study, the odds of early marriage among rural women were 4-folds higher compared to that among urban women. This finding is similar to the findings of studies conducted in Sudan, Bangladesh, and Serbia [52–54]. This could be explained by women who live in rural areas who may not know about the health, education, and economic impact of early marriage [55, 56]. Besides, they do not know where to go when their parents and/or their guardians violate their human rights declared by the national family law [55–57]. Consequently, women who resided in a rural part of the region are more prone to early marriage compared to women resided urban areas of the region.

The study revealed that women with no education level were 2.5 more likely to be married before the age of 18 compared to women who attended a higher education level. Similarly, the odds of early marriage among women who attended only primary education level was 2-folds higher compared to women who attended higher education level. These findings are similar to two studies conducted in Ethiopia that showed the educational level of women was found to be a significant predictor for early marriage [34, 58]. Moreover, other studies conducted in Malawi and Western Uganda also revealed that women's education level was the independent predictor for early marriage [27, 59]. This could be justified by the higher education level of women enabling them to be aware of their rights and empowering them to make their decision when to get marriage [2, 34, 60].

In this study, the odds of early marriage among women with the poorest household wealth index were 2.4 times higher compared to those among women with the richest household wealth index. This result is consistent with two studies conducted in Ethiopia and a study done in India [50, 58, 61]. This might be justified by the poorest families preferring early marriage to generate more income in the form of “macha” (i.e., male's family pay money and cattle for female's family) [35]. This is also supported by another study conducted in Ethiopia that revealed low economic status is one of the predisposing factors for early marriage [34, 55, 62]. Therefore, parents with low household income are still practicing early marriage as one of the alternative sources of income to relieve their economic problems. Consequently, women from the poorest families are prone to early marriage compared to women from the richest families.

Religious and cultural practices that defined puberty as the age of maturation consider a girl ready for marriage when she reaches puberty [63, 64] that deprives her decision-making autonomy. Similarly, in this study, the odds of early marriage among women whose age of the first marriage was decided by their parents was 3-folds higher compared to that among those whose age of first marriage was decided by themselves. This finding is similar to studies conducted in different settings [41, 65, 66]. In Ethiopia, particularly in Amhara region, there are deep-rooted harmful sociocultural practices in the community, whose females have no autonomy to choose their husband and to make decisions when to get married [34, 39, 67]. As a result, females are enforced to get married early without their involvement and consent.

## 5. Conclusion

The overall prevalence of early marriage among married reproductive age women in Amhara regional state is still high compared to the national prevalence.

After adjusting for covariates, women living in a rural part of the region, never attended formal education, being in the poorest and poorer household wealth status, and who never been part of decision-making when to get married were the independent predictors of early marriage in Amhara regional state.

Therefore, the regional government should give due attention to access to education and encourage women's decision-making power upon the age of marriage especially those residing in rural parts of the region. Moreover, the regional government should encourage women to participate in small-scale entrepreneurship to maximize their economic status. Additional community-based interventional studies are recommended to be carried out in the nearby future.

## 6. Strengths and Limitations of the Study

This study was based on the recent demographic health survey with a nationally representative large sample size. Furthermore, this study used multilevel logistic regression modeling to control the hierarchical nature of the EDHS data. Despite the above strengths, the study might have recall bias since the participants were asked the events that took place 5 years or more preceding the survey.

## Figures and Tables

**Table 1:** Sociodemographic characteristics of married reproductive-age women, Amhara regional state, Ethiopia, 2016.

Variable	Category	Weighted frequency	Percent
Residence	Urban	487	16.88
	Rural	2400	83.12
Religion	Orthodox	2,406	83.32
	Muslim	478	16.56
	Other+	3	0.21
Ethnicity	Amhara	2,778	96.21
	Oromo	61	2.13
	Agew	38	1.33
	Other++	10	0.33
Respondent's educational status	No education	1,938	67.10
	Primary	662	22.94
	Secondary	174	6.02
	Higher	113	3.94
Husband's educational status	No education	1,570	65.03
	Primary	540	22.35
	Secondary	144	5.97
	Higher	160	6.65
Respondent's occupation	Agriculture	1195	37.98
	Business	304	10.26
	Professional	87	3.67
	Daily laborer	195	6.75
	No job	1,081	38.52
	Other+++	25	2.81
Husband's occupation	Agriculture	1,784	73.91
	Business	179	7.42
	Professional	134	5.57
	Daily laborer	186	7.73
	Other+++	130	5.37
Wealth index	Poorest	434	15.03
	Poorer	577	19.97
	Middle	691	23.92
	Rich	655	22.70
	Richest	530	18.39
Who decide the marriage	Myself	435	15.09
	Parents	2405	83.28
	Other relatives/family	47	1.63
Media exposure	No	1889	65.43
	Yes	998	34.57

Other+: Catholics and protestants; Other++: Argoba and Kimant; Other+++: EDHS 2016 other category.

**Table 2:** Cross-tabulation of early marriage with independent variables in Amhara regional state, Ethiopia, 2016.

Variable	Category	Age at first marriage of women		
		≥18 years old	<18 years old	Total
Residence	Urban	244 (51.82%)	227 (48.18%)	487
	Rural	545 (22.58%)	1,871 (77.42%)	2,400
Religion	Orthodox Christian	643 (26.74%)	1,763 (73.26%)	2,406
	Muslim	133 (27.87%)	345 (72.13%)	478
	Other+	2 (57.39%)	1 (42.61%)	3
Ethnicity	Amhara	750 (26.99%)	2,028 (73.01%)	2,778
	Agew	7 (18.51%)	31 (%)	38
	Oromo	18 (29.96%)	43 (70.04%)	61
	Other++	3 (33.84%)	7(66.16%)	10
Who decides the marriage	Myself	254 (58.30%)	182 (41.70%)	436
	Parents	519 (21.56%)	1,886 (78.44%)	2,405
	Other relatives/family	17 (36.62%)	29 (63.38%)	46
Media exposure	No	443 (23.46%)	1,446 (76.54%)	1,889
	Yes	335 (33.59%)	663 (66.41%)	998
Women's educational status	No formal education	409 (21.13%)	1,529 (78.87%)	1,938
	Primary	195 (29.48%)	467 (70.52%)	662
	Secondary	103 (59.17%)	71 (40.83%)	174
	Higher	82 (72.33%)	31 (27.67%)	113
Husband's educational status	No formal education	319 (20.30%)	1,251 (79.70%)	1,570
	Primary	170 (31.42%)	370 (68.58%)	540
	Secondary	62 (43.34%)	82 (56.66%)	144
	Higher	104 (64.83%)	56 (35.17%)	160
Women's occupation	Agriculture	274 (22.96%)	921 (77.04%)	1195
	Professional	66 (76.21%)	21 (23.79%)	87
	Business	93 (30.75%)	211 (69.25%)	304
	Daily labor	61 (31.50%)	134 (68.50%)	195
	No job	286 (26.45%)	795 (73.55%)	1,081
	Other+++	8 (31.85%)	17 (68.15%)	25
Husband's occupation	Agriculture	383 (21.47%)	1,401 (78.53%)	1,784
	Professional	73 (54.78%)	61 (45.22%)	134
	Business	82 (45.83%)	97 (54.17%)	179
	Daily labor	57 (30.66%)	129 (69.34%)	186
	Other+++	59 (45.26%)	71 (54.74%)	130
Wealth index	Poorest	98 (22.64%)	336 (77.36%)	434
	Poorer	122 (21.22%)	455 (78.78%)	577
	Middle	157 (22.69%)	534 (77.31%)	691
	Rich	192 (29.29%)	463 (70.71%)	655
	Richest	220 (41.53%)	310 (58.47%)	530

Other+: Catholics and protestants; Other++: Argoba and Kimant; Other+++: EDHS 2016 other category.

**Table 3:** Community-level variance of two-level mixed-effect logit models predicting early marriage, Amhara regional state, Ethiopia, 2016.

Random effect	Null model	Full model
Community-level variance	0.49	0.44
ICC (%)	13	12
PCV (%)	Reference	10
Model fitness statistics (AIC)	3232.80	2442.02

**Table 4:** Multilevel multivariable logistic regression of the individual and community-related variables associated with early marriage, Amhara regional state, Ethiopia, 2016.

Variables	Categories	AOR	95% CI
Residence site	Urban (ref)		
	Rural	4.33	2.17–8.64^∗^

Wealth index	Richest (ref)		
	Richer	0.67	0.38–1.19
	Middle	0.86	0.45–1.65
	Poorer	1.38	1.16–4.83^∗^
	Poorest	2.37	2.19–7.82^∗^

Women's educational status	Higher (ref)		
	Secondary	0.88	0.19–4.02
	Primary	2.31	1.68–8.53^∗^
	No education	2.52	2.23–9.51^∗^

Husband education status	Higher(ref)		
	Secondary	1.88	0.59–6.01
	Primary	1.50	0.50–4.50
	No education	2.19	0.71–6.75

Media exposure	Yes(ref)		
	No	0.94	0.64–1.38

Decide at first marriage	My self (ref)		
	Parents	3.44	2.20–5.39^∗^
	Other relatives	1.67	0. 51–5.51

Husband occupation	Professional (ref)		
	Daily labor	0.73	0.24–2.28
	Business	0.33	0.11–1.03
	Agri/employee	0.71	0.25–1.98
	Other+++	0.51	0.17–1.57

Respondent occupation	Professional (ref)		
	Daily labor	0.98	0.21–4.47
	Business	1.24	0.66–2.34
	Agri/employee	1.09	0.27–4.42
	No job	1.44	0.37–5.63
	Other+++	2.37	0.31–17.67

ref: reference; ^∗^statistically significant variables at 95% Confidence Interval; Other+++: EDHS 2016 other category.

## Data Availability

The data used for this analysis is available from the Demographic and Health Survey (DHS) website (http://www.measuredhs.com).

## Abbreviations

AIC: Akaike Information Criterion
AOR: Adjusted Odds Ratio
CI: Confidence Interval
EAs: Enumeration areas
EDHS: Ethiopian Demographic and Health Survey
HIV/AIDS: Human Immune deficiency Virus/Acquired Immune Deficiency Syndrome
ICC: Intraclass correlation coefficient
PCV: Proportion Change in Variance
STIs: Sexually Transmitted Infections.
